# Multiple Small Bowel Cavernous Hemangiomatosis: Case Report and Literature Review

**DOI:** 10.3390/medicina60101664

**Published:** 2024-10-10

**Authors:** Francesca Ré, Salvatore Carrabetta, Eugenio Merlo, Pietro Bisagni

**Affiliations:** 1 General Surgery Department, Villa Scassi, 16149 Genova, Italy; francesca.re85@gmail.com (F.R.); salvatore.carrabetta@asl3.liguria.it (S.C.); 2 Pathology Department, Villa Scassi, 16149 Genova, Italy; eugenio.merlo@asl3.liguria.it; 3 General Surgery Department, ASST Lodi—Università Statale di Milano, 20122 Milano, Italy

**Keywords:** cavernous hemangiomatosis, gastrointestinal bleeding, small bowel, laparoscopy, case report

## Abstract

A 79 year old female individual presented to the hospital and complained of 1 month melena and anemia due to chronic gastrointestinal bleeding because of cavernous hemangiomatosis of the small bowel. After undergoing an initial video laparoscopic jejunal–ileal resection surgery 7 days after first hospitalization, given the persistence of anemia, she underwent laparotomic duodenojejunal resection surgery again 2 months later. Multiple cavernous hemangiomatosis is a rare vascular disease (7–10% of all benign small bowel tumors), and it often manifests with bleeding, which may be occult or massive; more rarely, it manifests with intestinal occlusion or perforation. Diagnoses often require the use of multiple radiological and endoscopic methods; video capsule endoscopy has significantly increased the diagnostic rate. The gold standard of treatment is surgical resection, whenever possible, balancing the need for radicality with the possible metabolic consequences of massive small intestine resections.

## 1. Introduction

Multiple cavernous hemangiomatosis of the small intestine is a rare disease. Hemangioma is a benign vascular origin tumor that can occur at any age. It may remain undiscovered but is occasionally found due to bleeding, which may be life-threatening or chronic, resulting in anemia [1,2,3]. Diagnosis is difficult and usually involves multiple radiological and endoscopic exams. Thanks to the use of video capsule endoscopy and deep enteroscopy, the percentage of patients obtaining a preoperative diagnosis has increased [4,5,6]. The gold standard of treatment is surgical resection, which is often the last diagnostic act together with laparotomic or laparoscopic exploration of the abdomen and definitive histological examination.

## 2. Case Presentation

**Figure 1:**
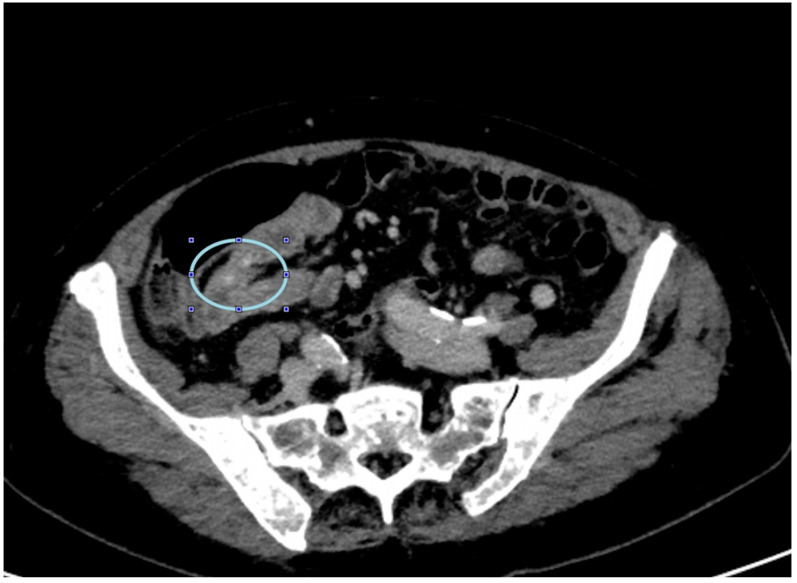
Angio CT contrast blushing in the proximal small bowel (first image).

**Figure 2:**
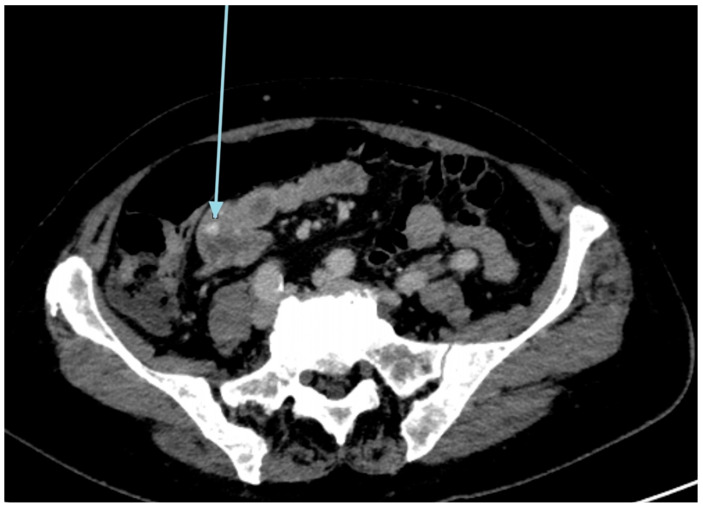
Angio CT contrast blushing in the proximal small bowel (late image).

**Figure 3:**
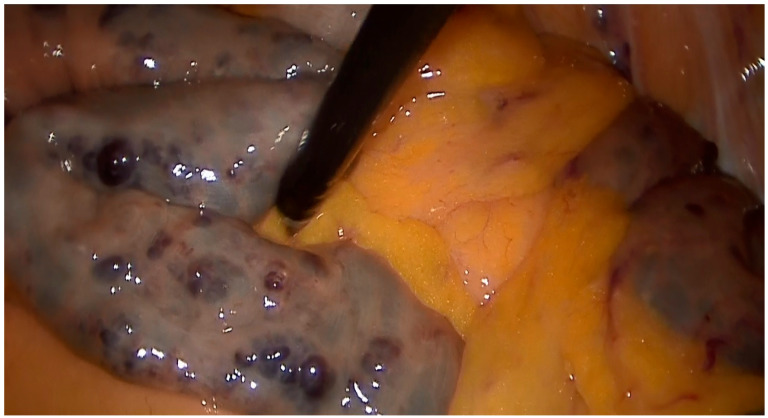
Laparoscopic first surgery intraoperative image showing multiple lesions in the proximal small bowel.

**Figure 4:**
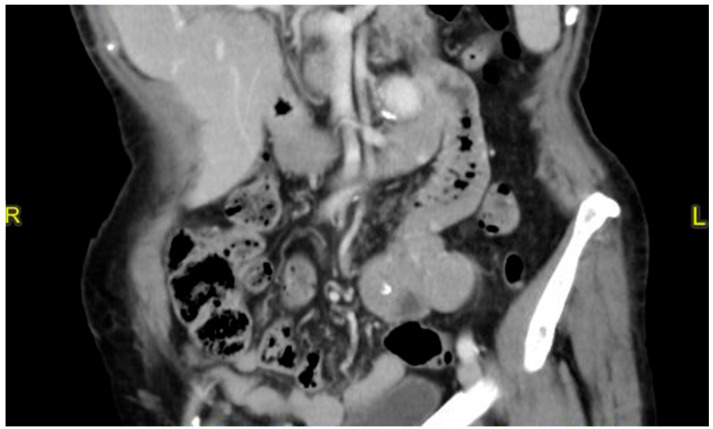

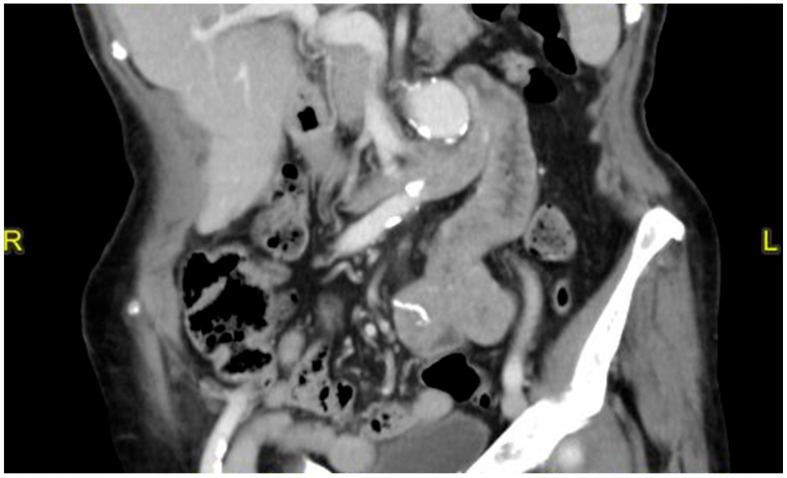
Angio CT showing multiple hyperdense endoluminal images at the fourh duodenum and first jejunal loop up to surgical anastomosis referable to contrast spillage.

**Figure 5:**
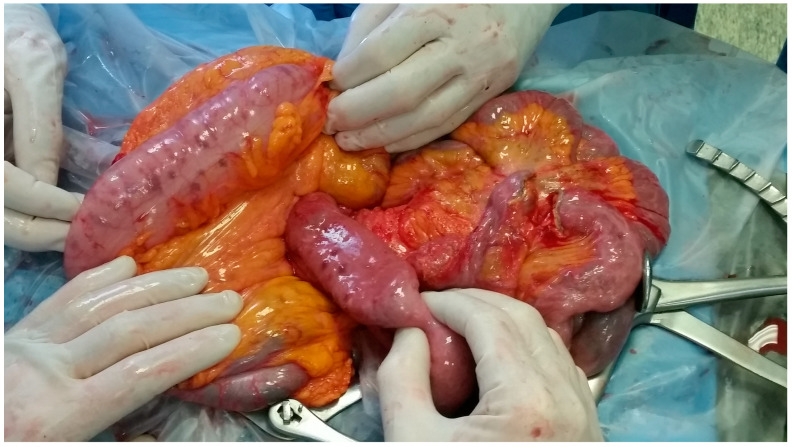
Intraoperative image of the second surgery showing multiple localization in the transverse colon and first jejunal loop.

**Figure 6:**
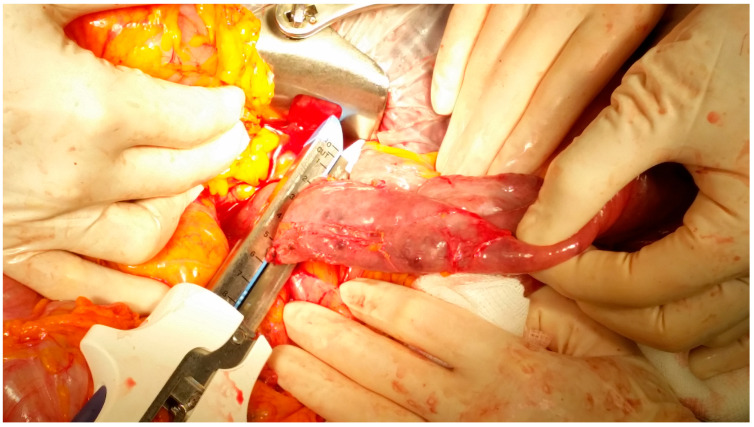
Proximal section of the duodenojejunal junction during the second surgery.

**Figure 7:**
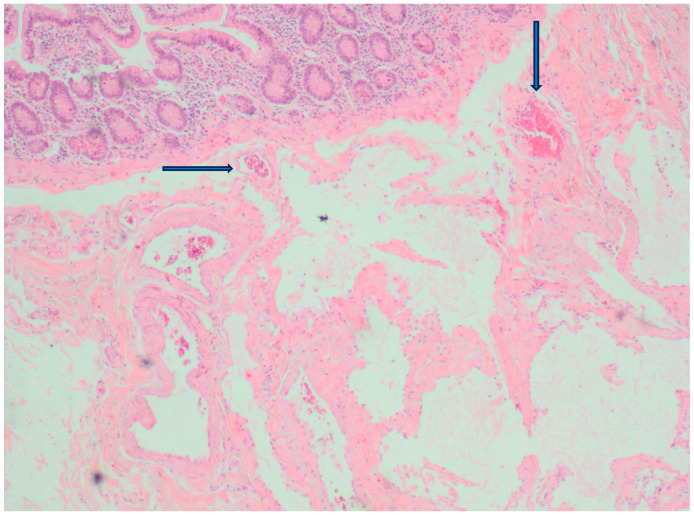
Hematoxylin eosin histology of the small bowel showing vascular submucosal sinus-like spaces containing blood separated by connective tissue; normal mucosal layer with crypts and villi is present too.

**Figure 8:**
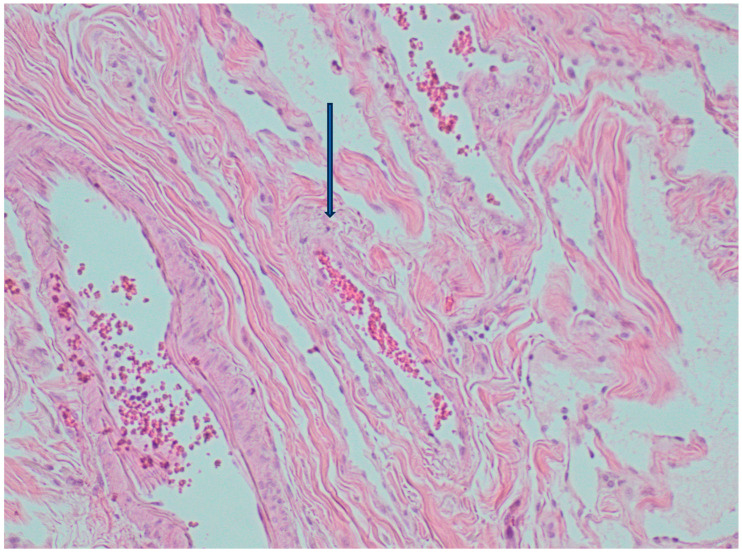
Hematoxylin eosin histology showing in detail endothelial lakes filled with blood and sinus-like spaces containing blood separated by connective matrix.

A 79-year-old lady was admitted to the emergency department for severe anemia (Hb 5.8 g/dL). The patient had been reporting melena for about a month. History included hypertension and oral anticoagulant therapy for a previous unspecified episode of transient amnesia and paroxysmal atrial fibrillation. The patient was admitted and underwent esophagogastroduodenoscopy and colonoscopy, both resulting within normal limits except for piceal stool in the right colon and terminal ileum. Abdominal contrast enhanced angio-CT exhibited sub-centimeter endoluminal blushing in the first bowel loop (Figure 1 and Figure 2). The diagnosis of arteriovenous malformation was reached by capsule endoscopy, which described important active bleeding at the very first jejunum, the origin of which, however, was not directly identified; no evidence of duodenal hemangiomas was found during EGDS or capsule endoscopy. In the remaining jejunum and initial ileum, the VCE showed numerous vascular wall abnormalities described as venous angiectasis and microvaricosities with some superficial erosions. Once the cause and site of the bleeding were presumably identified and Hb was brought to stable values of >9 g/dL by multiple transfusions, the patient underwent laparoscopic resection of the small intestine. During surgery, a blood content jejunum–ileum tract was resected, sparing the first 10 cm of jejunum, which was unharmed by active bleeding, and an extracorporeal latero-lateral double-layer manual anastomosis was performed through a small median incision (Figure 3). The procedure lasted 130 min. The patient remained hemodynamically stable during the procedure and did not require blood transfusions. The final histological examination revealed a 180 cm small bowel tract with multiple cavernous hemangiomatosis. The postoperative course was characterized by an initial recurrence of bleeding at the first oral refeeding instance that required blood transfusions and the resumption of parenteral nutrition. A subsequent attempt at oral feeding was uneventful, and she was discharged on postoperative day 20, with stable Hb values of >10 g/dL, regular oral feeding, and normochromic stools. The patient was referred to the Thrombosis Center for the optimization of anticoagulants assumption at this stage. Twelve days after discharge, she discontinued low-molecular-weight heparin and started taking cardio aspirin without reintroducing oral anticoagulants. Due to the persistence of chronic blood dripping and anemia requiring blood transfusions, 2 months later, the patient underwent EGDS, which reflected plausible angiomatous bleeding from the jejunum, which was not reachable using an endoscope. The patient then underwent CT angiography, which showed the appearance of multiple hyperdense endoluminal images at the IV duodenum and the jejunal loops up to the site of surgical anastomosis referable to contrast spillage, meaning ongoing bleeding, despite a stable Hb value (>7.5 g/dL) (Figure 4). After 75 days from discharge, the patient was then scheduled for a new surgery. The Hb value on admission was 6.8 g/dL due to persistent bleeding; thus, preoperatively, two units of blood were transfused. Exploratory laparotomy was performed, showing petechiae from the IVth duodenum and the proximal jejunum relative to the previous jejunum–ileal anastomosis. There was also evidence of a new appearance of petechiae at the level of the transverse and descending colon, which was not evident in preoperative investigations (Figure 5). The following course was decided for resection of the involved segment of the IVth duodenum and small bowel: manual double-layer latero-lateral duodenal–ileal anastomosis was performed, according to our usual anastomosis technique (Figure 6). The procedure lasted 155 min, with negligible blood loss. The final histological examination confirmed the diagnosis of localization of multiple cavernous hemangiomatosis of the small bowel. (Figure 7 and Figure 8). The postoperative course was uneventful, with no bleeding episodes, no need for transfusion, and the regular resumption of oral feeding. The patient was discharged on the eighth postoperative day, with Hb progressively increasing to 12.9 g/dL, in good clinical and hemodynamic condition, with normochromic stools and a free diet. At home, she is currently in good condition but experiencing diarrhea, presumably due to a short bowel, and she is compensated by dietary advice.

## 3. Results

Hemangioma of the small intestine is a rare benign vascular tumor [7,8]. The first case was reported in 1838 by Phillips [1,9]. Primary small bowel tumors are uncommon lesions, accounting for about 5% of all gastrointestinal cancers and, in 60–75% of cases, are benign [10,11]. Hemangiomas account for 7–10% of all benign small bowel tumors and 0.05% of all intestinal neoplasms [2,3,4,7,10,11,12,13,14]. They are fourth in frequency after adenoma, myoma, and fibroma [8,9]. 

Hemangioma is considered by many authors to comprise hamartoma [3,4,8,9,11,14,15,16,17]. Its etiopathogenesis has not yet been fully elucidated, and several hypotheses exist. According to some authors, hemangioma formation depends on altered angiogenesis mechanisms. Angiogenesis is tightly controlled by specific growth factors, and hemangioma could result from a local decrease in the inhibitors of these growth factors or a local increase in the growth factors themselves [4]. According to other authors, it would be an embryonic malformation resulting from an abduction of mesodermal tissue [16,17].

Hemangioma can occur in any part of the digestive tract [3,8,9,10]. There is no agreement regarding where it most frequently localizes: colon or small bowel [1,3,4,10,15]. Much more rarely, it is reported in the stomach, esophagus, and mesentery [3]. In any case, when localized in the small bowel, authors agree that the jejunal loop is frequently involved (46%) [2,3,4,5,10,12,14,18]. In our case, the occurrence localization turned out to be even more articulated, with an initial presence of multiple cavernous hemangiomas in jejunal–ileal loops and a later presence in the duodenum–jejunum and transverse and descending colon.

Hemangioma can present at any age: In fact, according to different researchers, the most frequent age of diagnosis might be between 5 and 25, in childhood or after the age of 30 [3,14]. In the literature, however, cases are reported in both children and the elderly. Our patient had multiple manifestation types prior to the introduction of oral anticoagulant therapy due to episodes of amnesia and paroxysmal atrial fibrillation, which probably facilitated bleeding; thus, the diagnosis was made at the manifestation of the issue at age 79.

Incidence is similar in both sexes, perhaps higher in women [3,10].

Hemangioma may be present in solitary or could more frequently presents as multiple localizations, which is the case reported here [7,10,11]. It may be associated with similar lesions in other viscera, mesentery, and skin, and may be one of the components of a syndromic manifestation [1,4,8,9,11,12,14,15]. No skin lesions were found in our case and, during surgery, it was verified that hemangiomatosis affected only the digestive tract and no other viscera or the mesentery.

The classification of hemangiomas has been revised several times. The most widely used one was defined by Abrahamson and Shandling in 1973, and it only includes three types of hemangiomas: capillary, mixed, and cavernous [3,4,7,10,11,12,14]. More recent classificatory revisions are those of the WHO in 2013 and ISSVA in 2014, which both still consider hemangioma to be a benign vascular tumor [3,19,20]. Macroscopically, hemangioma presents as a soft, sessile, or pedunculated polypoid structure that is blue, purple, or red in color; it varies in size from a few millimeters to a few centimeters, growing intraluminally or infiltrating the intestinal wall [3,4,10,12]. The most frequently observed type is cavernous hemangioma, which is composed of wide endothelial lakes filled with blood or sinus-like spaces containing blood separated by a connective tissue matrix [10,11,12,14]. Cavernous hemangioma is usually described as hemangioma that diffusely infiltrates the bowel wall such that it can extend to the mesentery, retroperitoneum, and pelvic wall, but it can also be more discrete in size as a submucosal nodule [2,3,9,14,17]. Capillary hemangioma, on the other hand, is described as a tuft of submucosal capillaries with intraluminal expansion that can form a mass (stalk-like mass) that can expand and infiltrate the intestinal wall leading to obstruction. Capillary hemangioma is often asymptomatic and localizes in the small intestine, right colon, appendix, and perianal region [1,17]. Upon immunohistochemistry, hemangioma is reactive for factor VIII and negative for keratin [3,14]. In a recent article, it has been reported that hemangioma exhibits a characteristic “endothelialized muscularis mucosae” reactive for endothelial markers CD31 and CD34 as well [2].

In contrast, angiodysplastic lesions (probably the most common cause of small bowel bleeding) are characterized by the presence of tortuous, thin-walled vessels in both the mucosal and submucosal layer of the small bowel [21].

Hemangioma may remain asymptomatic and be an occasional finding during other radiological exams, interventions, or autopsies [1,2,3]. Most likely, this would have been the case with our patient without oral anticoagulants. In fact, the most frequent manifestation of hemangioma is bleeding, which can present as a massive acute event or as chronic oozing leading to anemia (as in our case). Symptoms and signs may be melena, hematochezia, syncopal episodes, and iron deficiency (Table 1) [1,2,3,10,11,14,22].

We think that our patient had had some diffuse, small lesions for many years (maybe since birth); probably some lesions became larger and clinically evident after anticoagulants introduction and others grew after the first surgical procedure.

The diagnosis of small bowel hemangioma is not easy, not only as it is a rare condition but also because it does not have a typical age of presentation nor a typical location; moreover, it can manifest with different symptoms and signs. It usually requires multiple diagnostic, radiological, and endoscopic techniques (Table 1).

In our case, the pivotal presenting symptom was small bowel bleeding. Initial investigations of GI bleeding comprise gastroscopy and colonoscopy. The small bowel is difficult to explore, but after the introduction of video capsule endoscopy (VCE) and deep enteroscopy, most patients (about 75%) are now able to find the cause of their small bowel bleeding [4,5,6]. According to the latest update (2015) of the American College of Gastroenterology’s guidelines, VCE is considered a first-line method for small bowel exploration (full small bowel exploration in 79–90%; positive and negative predictive values of 94–97% and 83–100%, respectively) [4,6]. When a proximal small bowel lesion is suspected, push enteroscopy may be useful. It is effectively used for an endoscopic second look and, in the case of negative VCE, it is used upon suspicion of a proximal small bowel lesion [6]. For more distal lesions, deep enteroscopy techniques such as double-balloon enteroscopy (DBE), single-balloon enteroscopy (SBE), spiral enteroscopy (SE) and, finally, intraoperative enteroscopy (IOE) can be useful. 

The recommended radiological techniques for exploring the small intestine are CT, CT enterography (CTE), CT angiography (CTA), MR, MR enterography, conventional angiography, and tagged red blood cell scintigraphy [6,14]. Angiography can be used as a diagnostic and therapeutic tool in emergencies to control massive bleeding [6]. Finally, scintigraphy, usually used for the diagnosis of active bleeding (0.1–0.2 mL/min), has also been reported to be useful in cases of no actively bleeding hemangiomas identified by the blood stasis within them [22].

The treatment of choice for hemangioma is surgical resection whenever possible. As hemangioma is a benign disease, whether solitary or multiple, one should always try to be as conservative as possible. In case of multiple cavernous hemangiomatosis, the resection amount must be balanced with the extent of the disease and the metabolic consequences of short bowel. Focusing on definitive treatment, complete and radical excision of the involved intestinal tracts is mandatory; on the other hand, the avoidance of short bowel syndrome should be pursued too [8]. Therefore, in our case, during the first surgery, a decision was made to preserve the duodenum and proximal jejunum, but during the second surgery, new localizations at the colic level were found. Moreover, during the first surgery, evidence of bleeding was initially only observed in the resected jejunum–ileal tract (already quite extensive at 180 cm), and an attempt was initially made to avoid a duodenal resection and subsequent duodenum–ileal anastomosis—which certainly comprise greater complication risks in an elderly patient than a jejunum–ileal anastomosis. In the second surgery, because of persistence of anemia, we were obliged to carry out duodenal resection due to the presence of active bleeding in that area, but a decision was made to spare the transverse and descending colon, which contained new sites of the disease’s localization; they were not actively bleeding at the time of surgery and undetected upon preoperative investigations, and they were endoscopically reachable in case of bleeding. In fact, attempts at conservative medical or endoscopic treatment—which have, however, shown less efficacy than surgical resection in terms of bleeding control or recurrence—are burdened by even serious complications (e.g., massive bleeding and bowel perforation), and they are considered to be palliative [3,5,6,8,9,10,12,13,16,17]. In any case, endoscopy might be considered in elderly or unfit for surgery patients, as recently reported [39]. 

Laparoscopic complete abdominal cavity exploration allows for evaluation of the extent of the disease, leading to isolation and resection of the involved small bowel segment and intra or extraperitoneal anastomosis depending on local skills. This is a safe approach, and intraoperative enteroscopy can be associated with this method, using the light source of an enteroscope to carry out exploration of the suspected area [18]. It also has the advantages inherent to laparoscopy: less blood loss, fewer wound complications, decreased postoperative pain and shorter hospitalization [18,40].

## 4. Learning Points

- Small bowel cavernous hemangioma is rare and difficult to diagnose, involving a multidisciplinary team (radiologist, endoscopist, surgeons, and pathologist).
- Prompt surgical intervention is a key point in treating these patients.
- Laparoscopy might play an important role as a diagnostic and therapeutic tool.
- Intraoperative enteroscopy could be an option when the source of bleeding is unclear.

## 5. New Findings

- This case report emphasizes the possible multiple localizations and/or recurrence of bowel cavernous hemangioma.
- Although definitive treatment would involve complete removal of the disease, this goal must be balanced with the real risk of short bowel syndrome.

## 6. Conclusions

Multiple cavernous hemangiomatosis is a rare cause of small bowel bleeding. This diagnosis requires several exams in a multidisciplinary approach and should include VCE and possible deep enteroscopy. The therapy of choice is surgical resection, but this goal must be balanced with the risk of short bowel syndrome. The laparoscopic approach is feasible and safe.

## Figures and Tables

**Table 1:** Literature review of hemangioma case reports.

Author, Year	Age	Sex	Symptoms and Signs	Preop Radiological Examination	Diagnostic Examination	Lesion Location	Histology	Treatment
Cox, 1949 [7] USA	43	F	Abdominal pain, nausea, vomiting, melena, amenorrhea	-	Laparotomy	Ileum	Cavernous	Surgery
Grilli, 1989 [8] Italy	71	M	Abdominal pain, vomiting, occlusion	X-ray of the abdomen, barium enema,	Laparotomy	Ileum	Cavernous	Surgery
D’Armiento, 1989 [1] Italy	32	M	Abdominal pain, nausea, vomiting, sub occlusion, abdominal mass	X-ray of the abdomen, barium enema	Laparotomy	Ileum	Cavernous	Surgery
Kazama 2000 [23] Japan	64	M	Anemia	Barium enema, CT scan, MRI	Laparotomy	Jejunum	Cavernous	Surgery
Magnano 2005 [11] Italy	13	M	Fatigue, weakness, anemia		VCE, Laparotomy	Ileum	Cavernous	Surgery
Quentin 2007 [24] France	32	F	Hematochezia	CT scan	VCE, Laparotomy	Ileum	Cavernous	Surgery
Willert 2008 [25] Australia	19	M	Anemia	CT scan	VCE, balloon endoscopy	Jejunum, ileocecal valve	Cavernous	Endoscopic treatment
Pinho 2008 [12] Portugal	9	F	Fatigue, dizziness, anemia, melena		VCE	Ileum	Cavernous	Surgery
Chen 2009 [26] USA	23	M	Anemia		VCE	Ileum	Cavernous	Laparoscopic Surgery
Elias 2010 [27] USA	39	M	Anemia	CT scan	VCE, enteroscopy	Jejunum	Cavernous	Surgery
Huber 2012 [28] Germany	23	M	Melena, anemia		VCE, balloon endoscopy	Jejunum	Cavernous	Laparoscopic Surgery
Pera 2012 [29] Spain	15	M	Anemia, palpitation, fatigue		VCE, balloon endoscopy	Jejunum	Cavernous	Laparoscopic Surgery
Ersoy 2013 [30] Turkey	50	F	Hematemesis, melena		VCE, balloon endoscopy	Jejunum	Cavernous	Surgery
Fernandes 2014 [31] Spain	56	F	Hematochezia, syncope anemia	CT enterography	VCE	Ileum	Cavernous	Surgery
Bae 2015 [32] Korea	13	M	Nausea, dizziness, anemia		VCE	Jejunum	Cavernous	Surgery
Peng 2016 [33] China	47	M	Weakness, melena	CT scan	VCE, Laparotomy	Ileum	Cavernous	Surgery
Akazawa 2016 [5] Japan	56	F	Melena		VCE, balloon endoscopy	Jejunum	Cavernous	Laparoscopic Surgery
Ejtehadi 2017 [34] Iran	40	M	Fatigue, palpitation	Scintigraphy, CT enterography	Laparoscopy	Jejunum	Cavernous	Laparoscopic Surgery
Durer 2018 [35] USA	66	M	Anemia		VCE	Jejunum	Cavernous	Surgery
Hu 2018 [36] China	24	F	Melena, fatigue		VCE, laparoscopy	Ileum	Cavernous	Laparoscopic Surgery
Fu 2020 [37] China	5	F	Abdominal pain, vomiting, anemia	US, CT scan	Laparoscopy	Jejunum	Racemose	Laparoscopic Surgery
Baraldo, 2021 [38] Brazil	46	F	Abdominal distension, abdominal pain, anemia	CT scan	Laparotomy	Ileum	Cavernous	Surgery

## Data Availability

Restrictions apply regarding the availability of these data as they are not publicly available. However, the data are available from the corresponding author upon reasonable request.
